# Effects of Trimetaphosphate on Abiotic Formation and Hydrolysis of Peptides

**DOI:** 10.3390/life7040050

**Published:** 2017-11-30

**Authors:** Izabela K. Sibilska, Bingming Chen, Lingjun Li, John Yin

**Affiliations:** 1 Department of Chemical and Biological Engineering, Wisconsin Institute for Discovery, University of Wisconsin-Madison, 330 N. Orchard Street, Madison, WI 53715, USA; sibilska@wisc.edu; 2 Department of Chemistry, University of Wisconsin-Madison, 777 Highland Avenue, Madison, WI 53705, USA; bchen222@gmail.com (B.C.); lingjun.li@wisc.edu (L.L.)

**Keywords:** diglycine, drying, condensation, emergence, activation, *N*-phosphorylation, environment, HPLC, mass spectrometry

## Abstract

The primordial Earth probably had most of the factors needed for the emergence and development of life. It is believed that it had not only water, but also simple inorganic and organic materials. While studies since the 1950s on the origins of organic matter have established key roles for amino acids, conditions that would have promoted their condensation to make polymers, such as peptides or proteins, have yet to be fully defined. The condensation of amino acids in a water-rich environment is not thermodynamically favored. Therefore, the efficient formation of peptides requires the presence of a catalyst or the activation of a substrate. In living cells, the biosynthesis of proteins is assisted by enzymes and requires adenosine triphosphate (ATP), a relatively complex organic polyphosphate, which serves as an energy source. Outside the living organism, simpler inorganic polyphosphates can form active aminoacyl–phosphate anhydrides, which suggests the broader potential of phosphorus for enabling the polymerization of amino acids. However, this has yet to be demonstrated. To address this gap, aqueous solutions containing a simple dipeptide, diglycine, and a simple polyphosphate, trimetaphosphate, were dried, and reaction products were analyzed by high performance liquid chromatography and mass spectrometry (HPLC-MS). Different reaction environments, which were defined by the initial solution composition, pH, temperature, and incubation time, were found to affect the distribution and yield of products. Our results collectively provide strong evidence for reactions that both condense and hydrolyze peptides. It is noteworthy that the co-occurrence of reactions that form and cleave peptides are a central feature of Kauffman’s theory for the emergence of autocatalytic sets, which is a key step in the chemical origins of life.

## 1. Introduction

Phosphorus is an essential element of life, and presumably played a critical role in its origins. One hypothesis on the origins of phosphorus on Earth is that it was delivered, together with other inorganic and organic matter, by asteroids or meteorites [1,2]. It has been suggested that at the very early stages, phosphorus (III) served as a precursor for the formation of organophosphorus compounds [3,4]. As the young Earth cooled, phosphorus (III) might have been present as a phosphite species (HPO_3_^2−^), which has been identified on marine sediments of the Archean era [5]. In turn, phosphite ions might have been generated by oxidation from phosphides [6], which have been found in the form of various minerals in accessory phases in meteorites, lunar rocks, interplanetary dust particles, and comets [7]. Under current geochemical conditions, phosphorus exists mostly in a pentavalent state as orthophosphate ion (PO_4_^3−^). To consider what role phosphorus could have played in prebiotic peptide formation, it is useful to briefly review its current role in the highly evolved process of protein synthesis.

**Figure 1:**
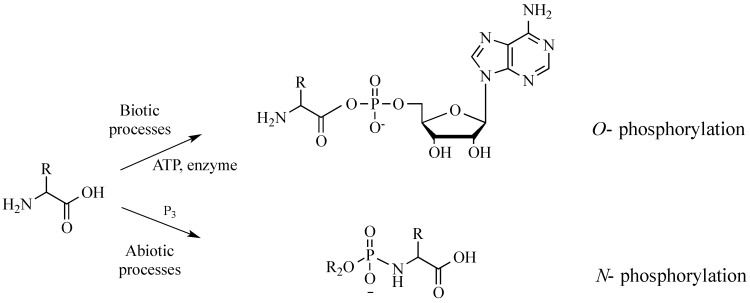
Possible pathways for the phosphate activation of amino acids.

In vivo protein synthesis involves the *O*-phosphorylation of the C-termini of amino acids, converting them to energy-rich amino acyl adenylates (Figure 1). Such activation of amino acids requires the enzyme aminoacyl tRNA synthetase, as well as adenosine triphosphate (ATP), a relatively complex organic polyphosphate. Mechanisms for the synthesis of ATP on the primitive Earth are an active topic of current debate [8]. In the absence of enzymes and ATP, amino acids can be activated using simpler, more elemental condensing agents, such as inorganic polyphosphates [9]. Such activation typically proceeds at the amino termini, where *N*-phosphorylation can release more free energy than the *O*-phosphorylation of catalyzed protein synthesis [10]. In nature, *N*-phosphoryl amino acids might represent a “living fossil” of prebiotic chemistries that have persisted; they are likely intermediates for many highly evolved enzymatic processes [11,12]. On the primitive Earth, inorganic polyphosphates such as trimetaphosphate might have served as a source of free energy. Trimetaphosphate (P_3_) could have been made directly from an acidic phosphate as a byproduct of volcanic processing [3,13]. More recently, the structural characterization of *N*-phosphoryl amino acids and the formation of the P–N bond have been elucidated by NMR [14] and IR [15] analysis, which have provided significant insight into the reaction mechanism [16]. Despite quite extensive studies on the activation of amino acids by polyphosphates, little effort has been devoted to study the subsequent dehydration (or condensation) reactions resulting in polypeptide formation.

Here, we tested the ability of trimetaphosphate (P_3_), a simple inorganic polyphosphate, to promote the synthesis of polypeptides during drying–heating processes from aqueous solutions at different temperatures and pH. Diglycine was selected as a reaction substrate because its α-carbon contributes minimal effects of steric and side-group chemistry. Further, the susceptibility of its single peptide bond to hydrolysis might be readily detected from the distribution of reaction products. Our studies indicate strong correlations between environmental conditions for peptide bond formation, its cleavage, and the final composition of the reaction mixture. We also demonstrate the stabilizing effect of polyphosphate against hydrolysis on the peptide bond, a characteristic that might have been important during prebiotic protein evolution.

## 2. Materials and Methods

### 2.1. Materials

All used chemicals were of analytical grade purity, purchased from Sigma Aldrich Chemical Company (St. Louis, MO, USA), and used without further purification. Reactions were carried out in 1.5 mL low-retention Eppendorf tubes. A standard modular heater with temperature control was used as a heat source (VWR). 

### 2.2. General Procedure for Drying-Induced Condensation of Diglycine in the Presence of Base

A solution of diglycine (6 mg; 50 μmol, 50 μL; 1 M aq. sol.) and NaOH (1.2 mg; 30 μmol, 30 μL, 1 M aq. sol.) in dd H_2_O (120 μL) was heated for 24 h at 70 °C open to the atmosphere. The dried pellet was then redissolved in 1 mL dd H_2_O and subjected to analysis by high performance liquid chromatography (HPLC).

### 2.3. General Procedure for Drying-Induced Condensation of Diglycine in the Presence of Trimetaphosphate

A solution of diglycine (6 mg; 50 μmol, 50 μL; 1 M aq. sol.) and sodium trimetaphosphate (4.6 mg; 15 μmol, 30 μL; 0.5 M aq. sol.) in dd H_2_O (120 μL) was heated for 24 h at 70 °C open to the atmosphere. The dried pellet was then redissolved in 1 mL dd H_2_O and subjected to analysis by HPLC.

### 2.4. General Procedure for Drying-Induced Condensation of Diglycine in the Presence of Trimetaphosphate and Base

A solution of diglycine (6 mg; 50 μmol, 50 μL, 1 M aq. sol.) an NaOH (1.2 mg; 30 μmol, 30 μL, 1 M aq. sol.) and sodium trimetaphosphate (4.6 mg; 15 μmol, 30 μL, 0.5 M aq. sol.) in dd H_2_O (90 μL) was heated for 24 h at 70 °C open to the atmosphere. The dried pellet was then redissolved in 1 mL dd H_2_O and subjected to analysis by HPLC.

### 2.5. IP-HPLC Analysis and Product Identification

A sample analysis was run using a Shimadzu Nexera XR IP-HPLC system fitted with a reversed-phase C18 column (Phenomenex Aeris XB-C18, 150 mm × 4.6 mm, 3.6 μL, Phenomenex Torrance, CA, USA). Samples were auto-injected in 10 μL aliquots (Shimadzu Nexera X2 Autosampler, Schimadzu Nakagyo-ku, Kyoto, Japan), and analysis was done in isocratic mode with a flow rate of 1 mL/min. The mobile phase was 50 mM KH_2_PO_4_ and 7.5 mM of C_6_H_13_SO_3_Na solution adjusted to pH 2.5 with H_3_PO_4_. The oligomeric products were detected at 195 nm, and the retention times were confirmed by comparison with pre-made standards containing glycine monomer and its oligomers. 

### 2.6. Product Quantification

The concentration of oligomer products was determined by the integration of absorbance values (195 nm) and calibration with commercially available standards (r^2^ ≥ 0.999). 

### 2.7. Mass Spectrometry

High resolution mass spectrometry (HRMS) analyses were performed on a MALDI-LTQ-Orbitrap XL (MALDI, matrix-assisted laser desorption/ionization) and an ESI Q Exactive HF Hybrid Quadrupole-Orbitrap (ESI, electrospray ionization) mass spectrometers (Thermo Fisher Scientific, Bremen, Germany) using positive ion mode. Prior to MS analyses, samples were mixed with equal volumes of acetic acid. For MALDI MS analysis, 1 μL of 2,5-dihydroxybenzoic acid (DHB) matrix (150 mg/mL in 49.95:49.95:0.1 = MeOH:H_2_O:formic acid) was mixed with 1 μL of analyte, and spotted onto a MALDI target plate. Full MS was acquired at *m*/*z* 100–800 with a mass resolution of 30,000 (at *m*/*z* 400). MS/MS analysis of each peak was performed using higher-energy collisional dissociation (HCD). Normalized collisional energy was optimized for each peak for optimum fragmentation efficiency. For ESI MS analysis, analyte was introduced into the mass spectrometer by direct infusion with a spray voltage of 3.5 kV. Full MS was acquired at *m*/*z* 80–1200 with a mass resolution of 30,000 (at *m*/*z* 200). MS/MS analysis was also performed using HCD with normalized collisional energy set to be 35. 

### 2.8. Diglycine Reaction Mixture Species Mass Identification

Triglycine: HRMS exact mass calculated for C_6_H_12_N_3_O_4_ [M + H]^+^ 190.0828, found 190.0837, HRMS exact mass calculated for C_6_H_11_N_2_O_4_Na [M + Na]^+^ 212.0647, found 212.0660; 

Tetraglycine: HRMS exact mass calculated for C_8_H_15_N_4_O_5_ [M + H]^+^ 247.1043, found 247.1060, HRMS exact mass calculated for C_8_H_14_N_4_O_5_Na [M + Na]^+^ 269.0862, found 269.0877; 

Pentaglycine: HRMS exact mass calculated for C_10_H_18_N_5_O_6_ [M + H]^+^ 304.1257, found 304.1295, HRMS exact mass calculated for C_10_H_17_N_5_O_6_Na [M + Na]^+^ 326.1077, found 326.1099; 

Hexaglycine: HRMS exact mass calculated for C_12_H_21_N_6_O_7_ [M + H]^+^ 361.1472, found 361.1367, HRMS exact mass calculated for C_12_H_20_N_6_O_7_Na [M + Na]^+^ 383.1291, found 383.1314;

Heptaglycine: HRMS exact mass calculated for C_14_H_24_N_7_O_8_ [M + H]^+^ 418.1686, found 418.1682, HRMS exact mass calculated for C_14_H_23_N_7_O_8_ [M + Na]^+^ 440.3688, found 440.3692;

Octaglycine: HRMS exact mass calculated for C_16_H_27_N_8_O_9_ [M + H]^+^ 475.1901, found 475.1901, HRMS exact mass calculated for C_16_H_26_N_8_O_9_ [M + Na]^+^ 497.1270, found 497.1268. 

## 3. Results

**Figure 2:**
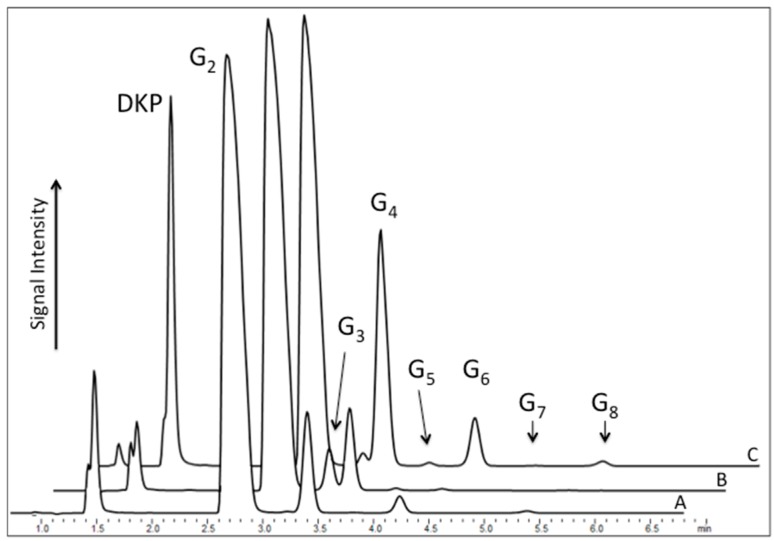
Ion pair high-performance liquid chromatography (IP-HPLC) chromatogram showing the formation of products obtained after heating diglycine for 24 h at 70 °C with trimetaphosphate (Curve A), NaOH (Curve B), and trimetaphosphate with NaOH (Curve C).

A typical reaction mixture consisted of 200 mM diglycine solution and sodium trimetaphosphate adjusted to pH ~ 9.5 with NaOH. The mixture was left open to the atmosphere while maintained at constant temperature for a specific time. Ambient humidity levels were not controlled during the course of the experiment. The resulting dry pellet was then redissolved in ultrapure water (18.2 Ω), and the mixture was analyzed by ion pair high-performance liquid chromatography (IP-HPLC). Analysis of the products after rehydration revealed several peaks, indicating the formation of different oligomers under different conditions (Figure 2). The identity of detected compounds was confirmed by comparison of their retention times with commercially available standards as well as mass spectrometric analysis. 

**Figure 3:**
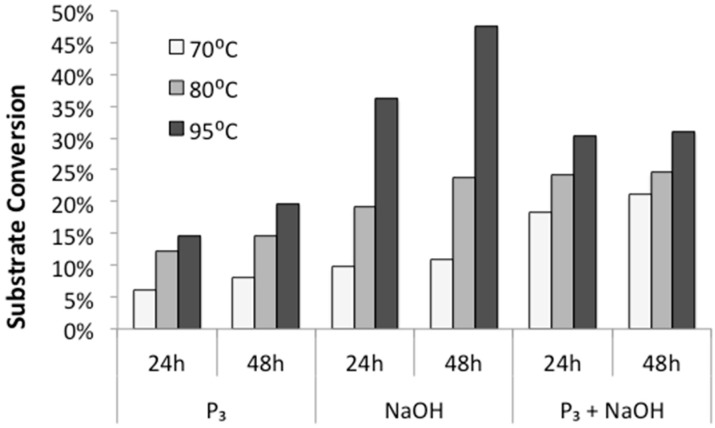
Extent of diglycine polymerization as a function of incubation time and temperature. Yields calculated from calibration curves (r^2^ ≥ 0.998).

First, we tested the influence of temperature and incubation time on polymerization rates. Various aqueous solutions of diglycine were incubated at 70 °C, 80 °C, and 95 °C, and open to the atmosphere for 24 h and 48 h to allow evaporation and dry-state reactions. HPLC analysis of redissolved solids showed that the conversion of diglycine into longer peptides increases both with higher temperatures and longer incubation time (Figure 3), which is in agreement with previous observations made by Borsook [17,18] on activated amino acids. In addition, we observed that the highest rates of substrate conversion into longer oligomers under mild temperatures (70 °C and 80 °C) were achieved from alkalized solutions of trimetaphosphate. When the reaction was carried out at elevated temperatures of 95 °C, the highest conversion of substrate (close to 50%) was observed in the mixtures of alkalized diglycine. 

Next, the ability of diglycine to polymerize was tested from the mixture consisting of the dimer and triphosphate in neutral solution (pH ~ 6.5), followed by the control reaction, which was an alkalized solution of the dipeptide. Here, HPLC profiles of analyzed mixtures revealed notably different results. When the alkalized solution of pH 9.5 permitted peptide hydrolysis, as reflected by the presence of the odd-numbered species Glyn, where *n* = 3, 5, 7, etc. (Figure 2 Curve B), the neutral reaction of diglycine with P_3_ yielded only even-numbered oligomers, indicating a suppression of amide bond cleavage (Figure 2 Curve A) by a stabilizing effect of the polyphosphate.

## 4. Discussion

**Figure 4:**
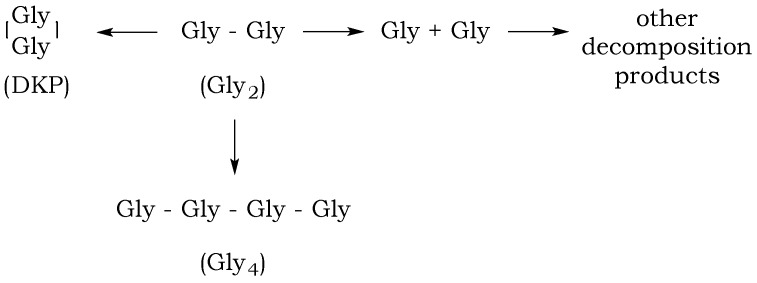
Possible reactions of diglycine in aqueous solution.

The dipeptide glycylglycine (Gly_2_) can undergo several reactions in aqueous solution: a linear condensation reaction, cyclization to diketopiperazine (DKP), peptide bond hydrolysis, and decomposition via decarboxylation and deamination (Figure 4). The rates of these reactions will depend on the pH of the aqueous media and the temperature and pressure of the reaction mixture, which will influence the distribution of the different species in solution over time [19]. Although the rate of the amide bond cleavage exponentially increases with increasing temperature, estimated standard molal thermodynamic properties of aqueous dipeptides and their constituent amino acids indicate that temperature increases correspond to the increased stability of peptide bonds relative to hydrolysis, resulting in a bias toward polymerization processes [19]. 

As expected, the conversion of diglycine to its longer oligomers increased with temperature (Figure 3), in agreement with the observation that for temperatures above 60 °C, the free energy barrier of peptide bond formation decreases, and therefore, bond formation between unprotected amino acids or peptides can be exergonic [1]. The extent of polymerization was likewise enhanced by incubation time (Figure 3), where samples typically appear visually dry by 6 h. This result indicates that the majority of the oligomer synthesis might take place in the absence of a bulk solvent in a dry solid state. 

**Figure 5:**
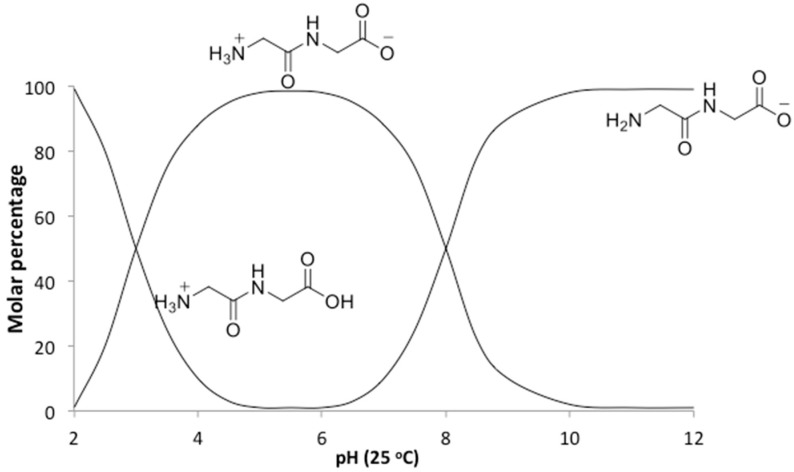
Diglycine species distribution as a function of pH at 25 °C.

**Scheme 1:**
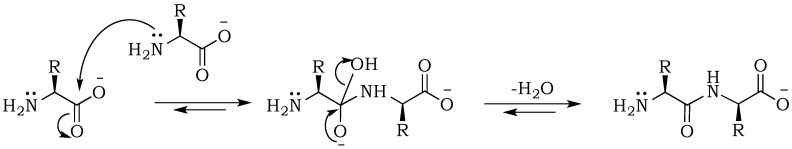
Mechanism of peptide bond formation in alkaline environment; R can be any side chain substituent.

In addition to the temperature, the solution pH would also be expected to affect the relative rates of polymerization and hydrolysis. An increase in pH from neutral to alkaline changes the ionic state of diglycine (Figure 5), which not only shifts the hydrolysis from acid-promoted through water-dependent neutral solutions to base-promoted hydrolysis assisted by OH^−^ ions, but more importantly affects nucleophilic and electrophilic relativities, which will be of vital importance for predicting rates of individual steps in the absence of enzymes. Peptides are formed from amino acids through an S_N_2 (Scheme 1) reaction that is initiated by the nucleophilic attack of an amino nitrogen on a carbonyl carbon, upon which electrons of the carboxyl double bond migrate to the oxygen atom in order to maintain the octet for the carbonyl atom. This step results in the formation of a tetrahedral intermediate. At the same time, the positive charge on the nitrogen is neutralized by proton migration to the negatively charged oxygen. Condensation is followed by the removal of the hydroxyl group and reformation of the double bond. This step starts with electrons moving back from the oxygen atom into the double bond, and expelling the –OH group from the carbonyl-carbon. Although the hydroxy moiety is not a good leaving group, this process is facilitated by the energetically favorable formation of the carbon-oxygen double bond and water removal during the drying process [20]. The enhancement of the reaction at alkaline pH can be explained mainly by the electrostatic properties of the substrates, as well as an increase of the nucleophilicity of the amine resulting from its deprotonation [21]. At pH ~ 10 diglycine is present mostly in an anionic state Gly_2_^−^, with only a small fraction of the zwitterionic Gly_2_^±^ form (Figure 5). Since Gly_2_^−^ has a deprotonated amino group with higher nucleophilicity, it has a great advantage for attacking the carbonyl carbon on the negatively charged C-terminus of a neighboring zwitterionic Gly_2_^±^ and anionic Gly_2_^−^, promoting peptide formation. This scenario assumes that the reaction medium is an ideal electrolyte solution, where only H^+^ transfers between the substrate, and dissolved species are considered. However, our reaction medium is neither ideal nor a solution, so other plausible mechanisms will need to be elucidated by in situ experimental and computational studies. 

**Scheme 2:**
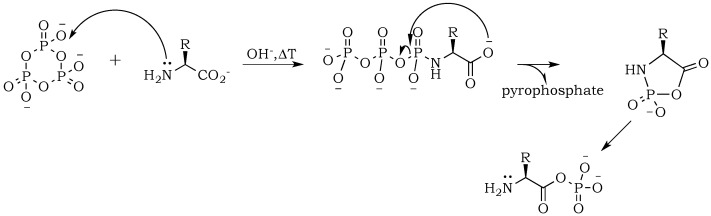
Mechanism showing the formation of a five-membered active intermediate with intramolecular phosphate transfer in alkaline solution; R can be any side chain substituent.

**Scheme 3:**
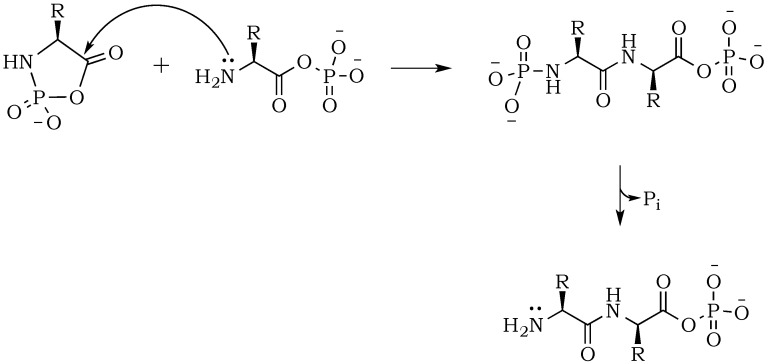
Mechanism showing a nucleophilic attack of free amine resulting in peptide bond formation in alkaline solution; R can be any side chain substituent.

The introduction of trimetaphosphate (P_3_) into the reaction mixture allows the formation of a highly active intermediate as a phosphoryl–carboxyl mixed anhydride (Scheme 2) [22]. Formation of the *N*–P bond is now possible, and it starts with the deprotonation of the α-amino group, followed by the nucleophilic attack of –NH_2_ at phosphorus in P_3_, and fragmentation of the resulting adduct with the elimination of pyrophosphate [14,23]. The fragmentation may proceed through a cyclic intermediate, during which migration of the phosphate residue from *N*– to *O*– is possible. However, if the formation of a five-membered ring is restricted by substrate conformations or the low proximity of reactive sites, then the probability of an intermolecular transfer becomes more plausible (Scheme 3) [24]. Here, the nucleophilic attack of a free amine group from one amino acid or peptide on the phosphate-activated carboxyl carbon yields a new peptide product. 

**Figure 6:**
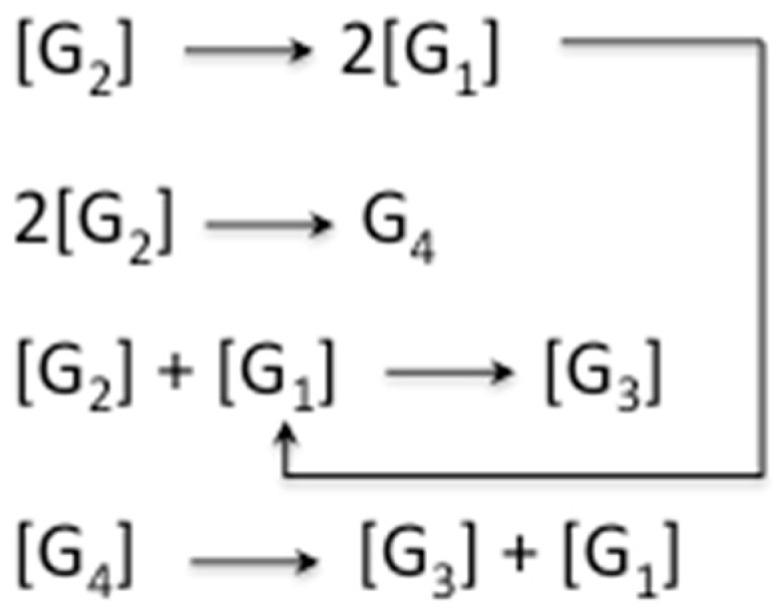
Possible reactions leading directly to formation of the triglycine Gly_3_.

**Scheme 4:**
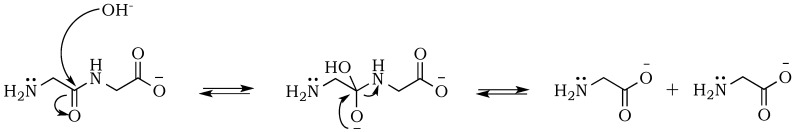
Base-promoted hydrolysis of the peptide bond.

**Scheme 5:**
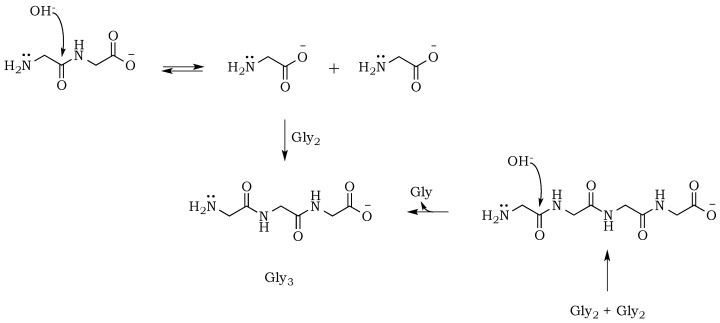
Proposed pathways leading to triglycine (Gly_3_) formation in alkaline solution in the absence of trimetaphosphate (P_3_).

**Scheme 6:**
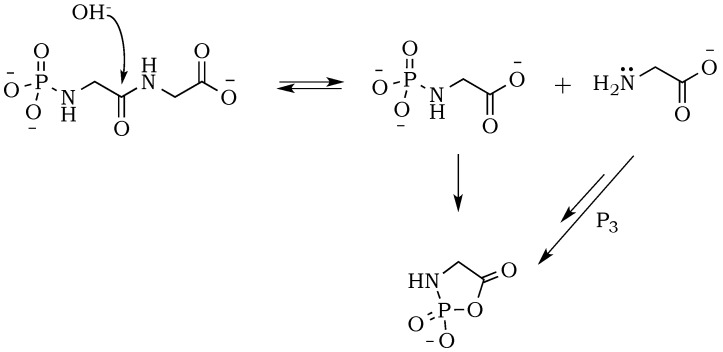
Possible mechanism leading to formation of cyclic mixed anhydride from *N*-phosphorylated diglycine in an alkaline solution in the presence of trimetaphosphate (P_3_).

**Scheme 7:**
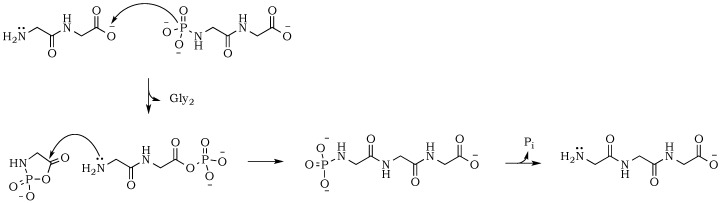
Proposed mechanism of diglycine reaction with cyclic mixed anhydride resulting in triglycine (Gly_3_) formation through intermolecular phosphate transfer.

The propensity of polymerization reactions to be favored at high pH can be explained by electrostatic interactions influencing the ionic character of the reacting molecules. Moreover, an activating character of phosphate is clearly visible if we compare diglycine conversion to higher oligomers in alkalized solutions in the presence vs. absence of trimetaphosphate (Figure 3). Although the amide bond is relatively unreactive, one issue connected to the condensation is the parallel and inevitable progression of hydrolysis (Scheme 4). In alkalized diglycine mixtures (lacking phosphate), the presence of odd-numbered species Gly*_n_* (where *n* = 3, 5, 7, etc.), along with the expected series of even-numbered oligomers (for *n* = 2, 4, 6, etc.), are consistent with previous observations suggesting dynamic combinatorial processes that form and break the C–N bond [25]. In this way, any odd-numbered species could be a product of at least two different pathways when condensation and hydrolysis proceed as two parallel events. For example, triglycine could be a product of either a diglycine condensation with glycine, or the hydrolysis of any of the higher oligomers, such as tetraglycine (Scheme 5, Figure 6). However, while analyzing products formed in the presence of the phosphate, we noted a suppression of hydrolysis. Drastically lower concentrations of tri- and pentaglycine indicate that phosphates must have a stabilizing effect on the peptide bond, most likely by increasing the hydrogen interaction of the amide proton with lone pair electrons localized on the phosphate oxygen. Since the reaction mixture has an alkaline environment, base-promoted hydrolysis is still possible, but it clearly proceeds with lower yields (Scheme 6). Now, the formation of an odd numbered oligopeptide (Scheme 7) would involve a five-membered phosphoryl-carboxyl mixed anhydride intermediate, which can be attacked by any available free amino group (not protonated and not involved in a P–N bond). This step proceeds regardless of whether the C-terminus of the attacking nucleophile has its oxygen in a phosphate bond or it carries a lone pair of electrons. Also, the product of this reaction could be either further elongated by condensation, or cleaved by hydrolysis reactions, respectively. 

**Scheme 8:**
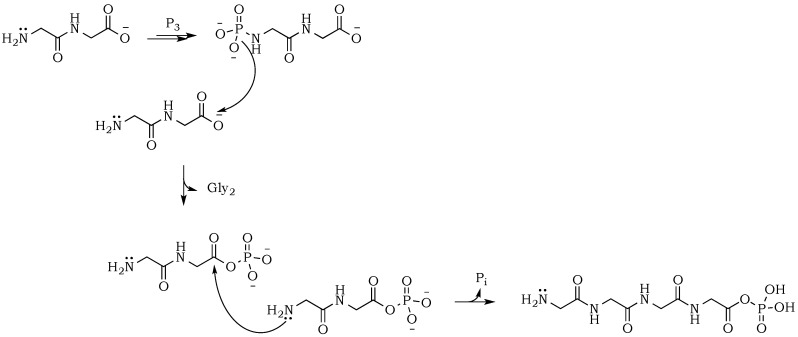
Proposed mechanism of diglycine condensation in the presence of trimetaphosphate (P_3_) from a neutral solution.

To check whether the peptide bond is stabilized against hydrolysis by phosphate, we performed a drying-induced polymerization of diglycine in neutral mixtures of trimetaphosphate, and noticed the complete inhibition of the peptide bond hydrolysis (Figure 2A). However, the conversion of diglycine was slightly lower when compared with alkaline reactions mixtures, which might be attributed to the higher level of zwitterion form and its associated lower nucleophilicity of the amino group. Moreover, under neutral pH, the carbonyl group activation might be able to proceed only through a potentially rate-limiting intermolecular reaction of the migrating phosphate from *N*– to *O*– (Scheme 8). This stabilizing effect of phosphate moiety was not reported before, either in works of Yamagata [26] or Yamanaka [22]. 

Taken together, these results indicate that one can have a significant environmental influence over the processes that determine the final composition of the reaction mixture. If the reaction environment allows the simultaneous formation and hydrolysis of the amide bond, starting from different monomers species, then a potentially much broader diversity of paths is made available for the production of each heteropolymer. The combinatorial possibilities, which have been worked out, indicate a key role for hydrolysis, in tandem with condensation, to enable the emergence and closure of sets of oligopeptides that collectively and cooperatively self-replicate [27]. This simple and artificially created environment, with control over temperature, pH, and the presence of an inorganic polyphosphate, will serve as a platform for creating more diversified chemical settings. If at the early stages of chemical evolution, one molecule could perform only one action, there might have been a need for many players present in the system, which could over time have combined and evolved into multitasking macromolecules. 

How information-rich highly functional polymers could arise and persist in the absence of pre-existing templates or precursors is still a key gap in our understanding of prebiotic chemistry and the chemical origins of life. Here, we have added to our understanding of the chemical evolution of *N*-phosphoryl amino acids by showing how they can influence condensation and hydrolysis reactions during the drying-induced synthesis of polypeptides. Expansion of this work will employ more diverse starting materials under abiotically relevant defined conditions. Use of different monomers will enable the synthesis of heteropolymers that encode information about their physical and chemical environments. Such products may eventually catalyze reactions that contribute to their own synthesis and persistence. 

## References

[1] Altwegg K., Balsiger H., Bar-Nun A., Berthelier J.-J., Bieler A., Bochsler P., Briois C., Calmonte U., Combi M.R., Cottin H. (2016). Prebiotic chemicals—Aminio acid and phosphorus—In the coma of comet 67P/Churyumov-Grasimenko. Sci. Adv..

[2] Cobb A.K., Purditz R.E., Pearce B.K.D. (2015). Nature’s Starships II: Simulating the Synthesis of Amino Acids in Meteorite Parent Bodies. Astrophys. J..

[3] Pasek M.A. (2007). Rethinking early Earth phosphorus geochemistry. Proc. Natl. Acad. Sci. USA.

[4] Bryant D.E., Greenfield D., Walshaw R.D., Johnson B.R.G., Herschy B., Smith C., Pasek M.A., Telford R., Scowen I., Munshi T. (2013). Hydrothermal modification of the Sikhote-Alin iron meteorite under low pH geothermal environments. A plausibly prebiotic route to activated phosphorus on the early Earth. Geochim. Cosmochim. Acta.

[5] Pasek M.A., Harnmeijer J.P., Buick R., Gull M., Atlas Z. (2013). Evidence for reduced phosphorus species in the early Archean ocean. Proc. Natl. Acad. Sci. USA.

[6] Kee T.P., Bryant D.E. (2006). Direct evidence for the availability of reactive, water soluble phosphorus on the early Earth. H-Phosphonic acid from the Nantan meteorite. Chem. Commun..

[7] Pasek M.A., Lauretta D. (2008). Extraterrestrial Flux of Potentially Prebiotic C, N and P to the early Earth. Orig. Life Evol. Biosph..

[8] Stairs S., Nikmal A., Bucar D.K., Zheng S.L., Szostak J.W., Powner M.W. (2017). Divergent prebiotic synthesis of primitive and 8-oxo-purine ribonucleotides. Nat. Commun..

[9] Hulshof J., Ponnamperuma C. (1976). Prebiotic condensation reactions in an aqueous medium: A review of condensing agents. Orig. Life.

[10] Goldwhite H. (1981). Introduction to Phosphorus Chemistry.

[11] Matthews H.R. (1995). Protein kinases and phosphates that act on histidine, lysine, or arginine residues in eukaryotic proteins: A possible regulator of the mitogen-activated protein kinase cascade. Pharmacol. Ther..

[12] Todd A. (1959). Some aspects of phosphate chemistry. Proc. Natl. Acad. Sci. USA.

[13] Yamagata Y., Watanabe H., Saitoh M., Namba T. (1991). Volcanic producton of polyphosphates and its relevance to prebiotic evolution. Nature.

[14] Li Y., Yin W., Zhao Y. (1992). Phosphoryl group participation leads to peptide formation from *N*-phosphorylamino acids. Int. J. Protein Res..

[15] Inoue H., Baba Y., Furukawa T., Maeda Y., Tsuhako M. (1993). Formation of Dipeptide in the Reaction of Amino Acids with cyclo-Triphosphate. Chem. Pharm. Bull..

[16] Gao X., Deng H., Tang G., Liu Y., Xu P., Zhao Y. (2011). Intramolecular phosphoryl transfer of *N*-phosphoryl amino acids. Eur. J. Org. Chem..

[17] Boorsook H. (1953). Peptide bond formation. Adv. Protein Chem..

[18] Tattersall R., Flegmann A.W. (1979). Energetics of peptide bond formation at elevated temperatures. J. Mol. Evol..

[19] Qian Y., Engel M.H., Macko S.A., Carpenter S., Deming J.W. (1993). Kinetics of peptide hydrolysis and amino acid decomposition at high temperature. Geochim. Cosmochim. Acta.

[20] Forsythe G.J., Yu S., Mamajanov I., Grover M.A., Krishnamurtyhy R., Fernandez F.M., Hud N.V. (2015). Ester-mediated amide bond formation driven by wet-dry cycles: A possible path to polypeptides on the prebiotic Earth. Angew. Chem. Int. Ed..

[21] Sakata K., Kitadai N., Yokoyama T. (2010). Effect of pH and temperature in dimerization rate of glycine: Evaluation of favorable environmental conditions for chemical evolution of life. Geochim. Cosmochim. Acta.

[22] Yamanaka Y., Inomata K., Yamagata Y. (1988). Condensation of oligoglycines with trimeta- and tetrametaphosphate in aqueous solutions. Orig. Life Evol. Biosph..

[23] Chung N.M., Lohrmann R., Orgiel L.E. (1971). The mechanism of the trimethaphosphate- induced peptide synthesis. Tetrahedron.

[24] Lohrmann R., Orgiel L.E. (1973). Prebiotic activation processes. Nature.

[25] Rodriguez-Garcia M., Surman A.J., Cooper G.J.T., Saurez-Marina J., Hosini Z., Lee M.L., Cronin L. (2015). Formation of oligopeptides in high yields under simple programable conditions. Nat. Commun..

[26] Yamagata Y., Inomata K. (1997). Condensation of glycylglycine to oligoglycines with trimethaphosphate in aqueous solutions. II: Catalytic effect of magnesium ion. Orig. Life Evol. Biosph..

[27] Kauffman S.A. (1986). Autocatalytic sets of proteins. J. Theor. Biol..

